# Intraduodenal wall recurrence 11 years after distal pancreatectomy for pancreatic ductal adenocarcinoma

**DOI:** 10.1007/s12328-025-02141-y

**Published:** 2025-05-16

**Authors:** Mikkaichi Ko, Junichi Kaneko, Rika Fujino, Hisamichi Yoshii, Hideki Izumi, Tomoko Sugiyama, Takuma Tajiri, Tomoko Hanashi, Masaya Mukai, Hiroyasu Makuuchi

**Affiliations:** 1 Department of Gastrointestinal Surgery, Hepato-Biliary-Pancreatic Surgery, Tokai University, Hachioji Hospital, 1838 Ishikawa, Hachioji, Tokyo 192-0032 Japan; 2 Department of Diagnostic Pathology, Tokai University Hachioji Hospital, Hachioji, Tokyo Japan; 3 Department of Gastrointestinal Surgery, Tokai University Tokyo Hospital, Hachioji, Tokyo Japan

**Keywords:** Intraduodenal wall recurrence, Late recurrence, Pancreatic ductal adenocarcinoma

## Abstract

Although late recurrence of pancreatic ductal adenocarcinoma (PDAC) is rare, it has been observed in local or regional lymph nodes, as well as in the liver, lung, or peritoneum. We report the first case of intraduodenal wall recurrence 11 years after distal pancreatectomy for PDAC.

## Introduction

Pancreatic ductal adenocarcinoma (PDAC) is a refractory cancer; recurrences frequently occur within the first 2 years after surgery, with up to 80% recurring within 5 years of resection [1]. Late recurrence of PDAC is rare but has been observed in local or regional lymph nodes, as well as in the liver, lung, or peritoneum [2]. Here, we report the first case of intraduodenal wall late recurrence of PDAC more than a decade after distal pancreatectomy.

## Case report

We report the case of a Japanese woman who presented with vomiting and abdominal distension at 74 years of age. She was referred to our hospital after an esophagogastroduodenoscopy showed duodenal stenosis.

**Fig. 1:**
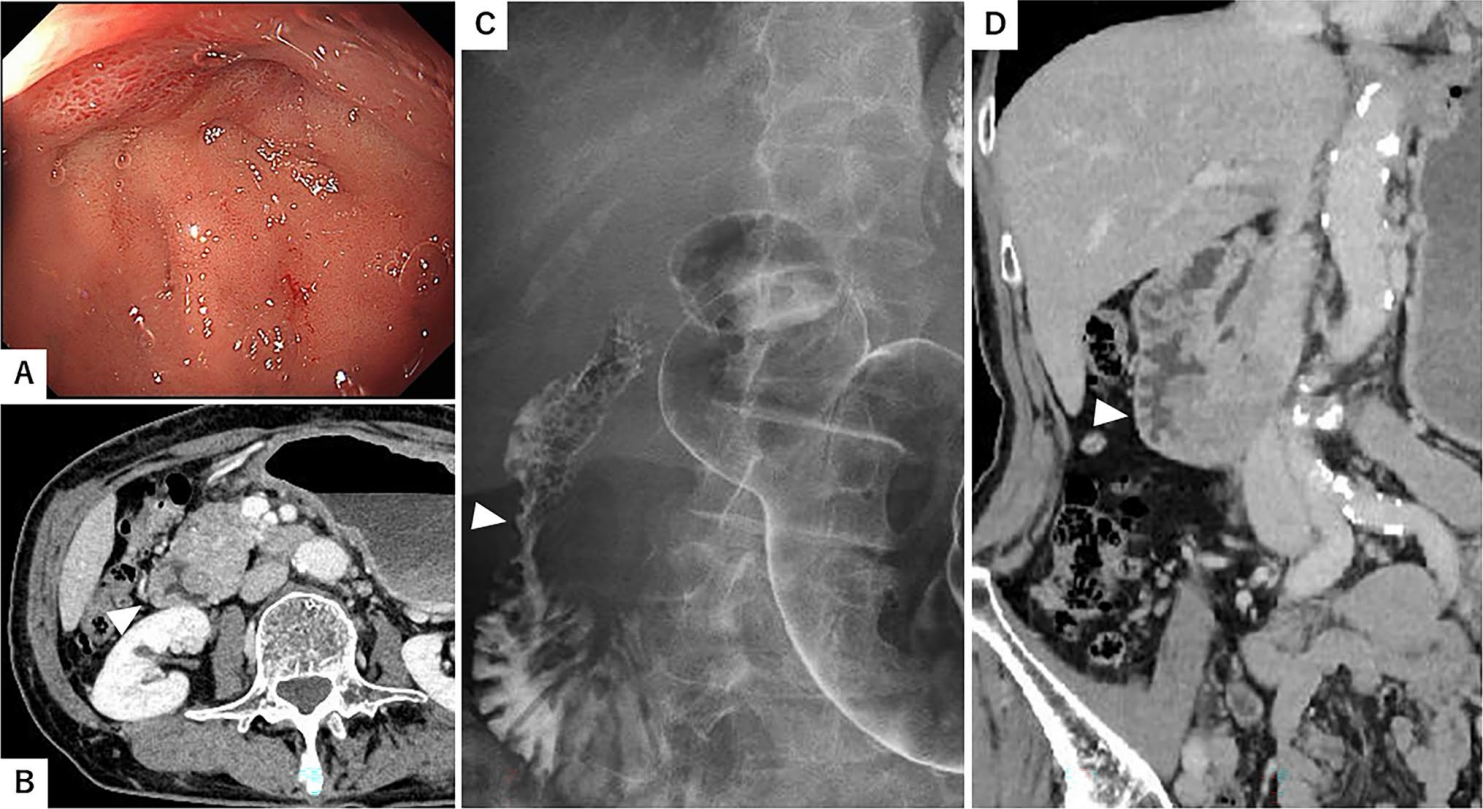
Esophagogastroduodenoscopy showed circumferential stenosis from the bulb of the duodenum that did not permit passage of the scope (**A**). The computed tomography scan showed thickening of the circumferential duodenal wall (**B**, **D**, triangle). Upper gastrointestinal series showed circumferential stenosis in the descending part of the duodenum (**C**, triangle)

Her height and weight were 156 cm and 42 kg, respectively. Her abdomen was soft and flat with no tenderness. Serum albumin and carbohydrate antigen (CA 19–9) levels were 3.4 g/dl and 69.3 U/ml, respectively, with no other abnormal data. Esophagogastroduodenoscopy showed circumferential stenosis from the duodenal bulb that would not permit passage of the scope (Fig. 1A). Although no ulcer was present, the biopsy revealed chronic inflammatory changes with no evidence of cancer cells. A computed tomography (CT) scan revealed thickening of the circumferential duodenal wall (Fig. 1B, D). Upper gastrointestinal series showed circumferential stenosis in the descending part of the duodenum (Fig. 1C). Positron emission tomography-CT revealed a weak hot spot in the descending duodenum.

The patient was diagnosed with suspected duodenal or remnant pancreatic cancer, and informed consent was obtained. A remnant pancreaticoduodenectomy was performed as part of a total pancreatectomy. The operation time was 2 h 50 min, and the estimated blood loss was 395 ml.

**Fig. 2:**
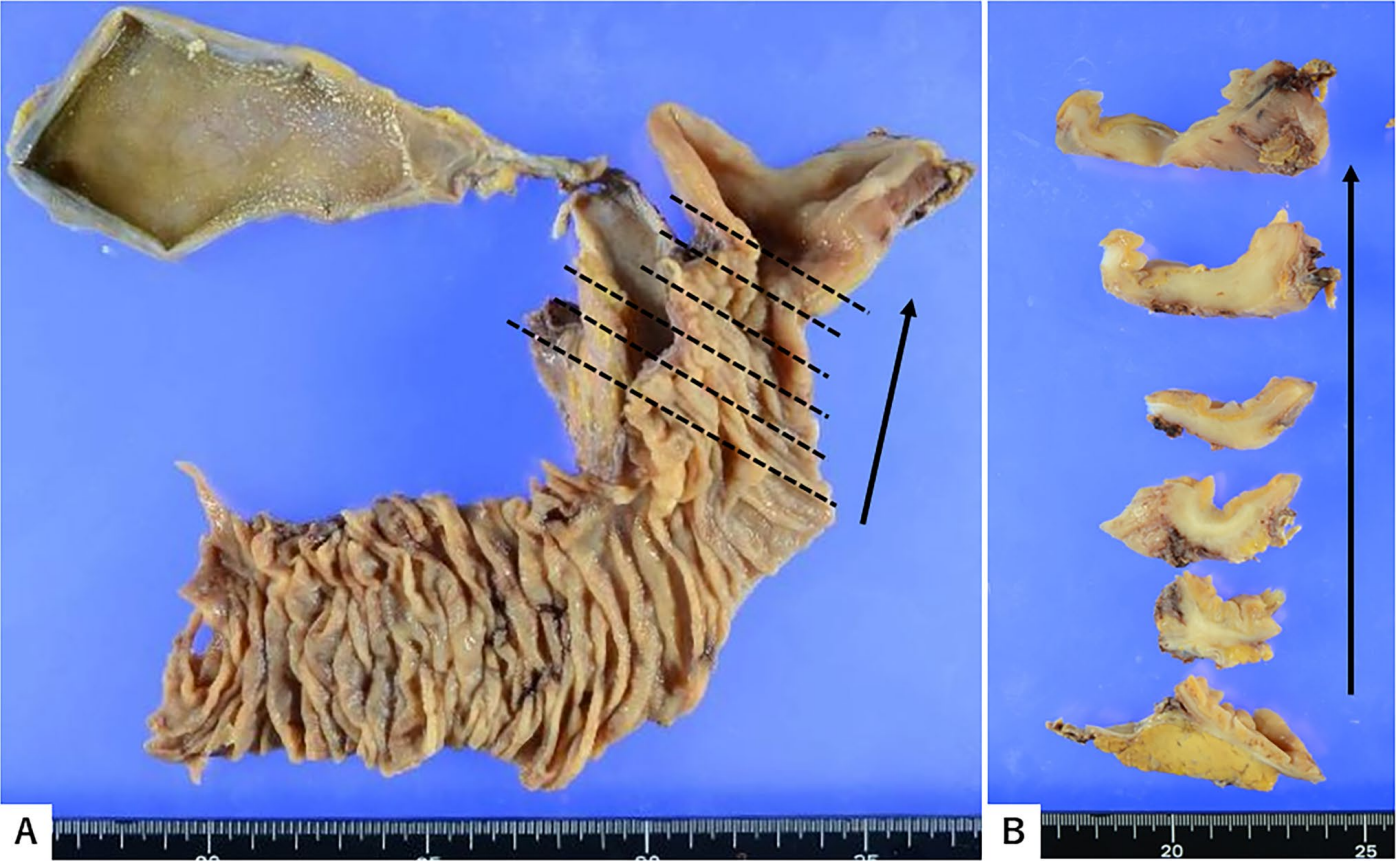
Surgical specimen from pancreatoduodenectomy and macroscopic cross-sections of the pancreatic duct. Macroscopically, there was no obvious mucosal lesion or ulcer in the duodenum (**A**). Note the firm, whitish lesion in the deeper submucosal layer of the duodenal wall in the cross sections (**B**)

Although macroscopic inspection of the biopsy specimens revealed no obvious mucosal lesion or ulcer in the duodenum (Fig. 2A), cross-sectional analysis identified a firm, whitish area within the deeper submucosal area (Fig. 2B), suggesting malignancy. Histologically, a moderately differentiated tubular adenocarcinoma (20 × 18 mm in diameter) was identified in the duodenal wall. Metastasis was observed in lymph nodes 13a and 17a, in association with a few lymphovascular and moderate nerve invasion were shown. The surgical margins were free of cancer cells.

**Fig. 3:**
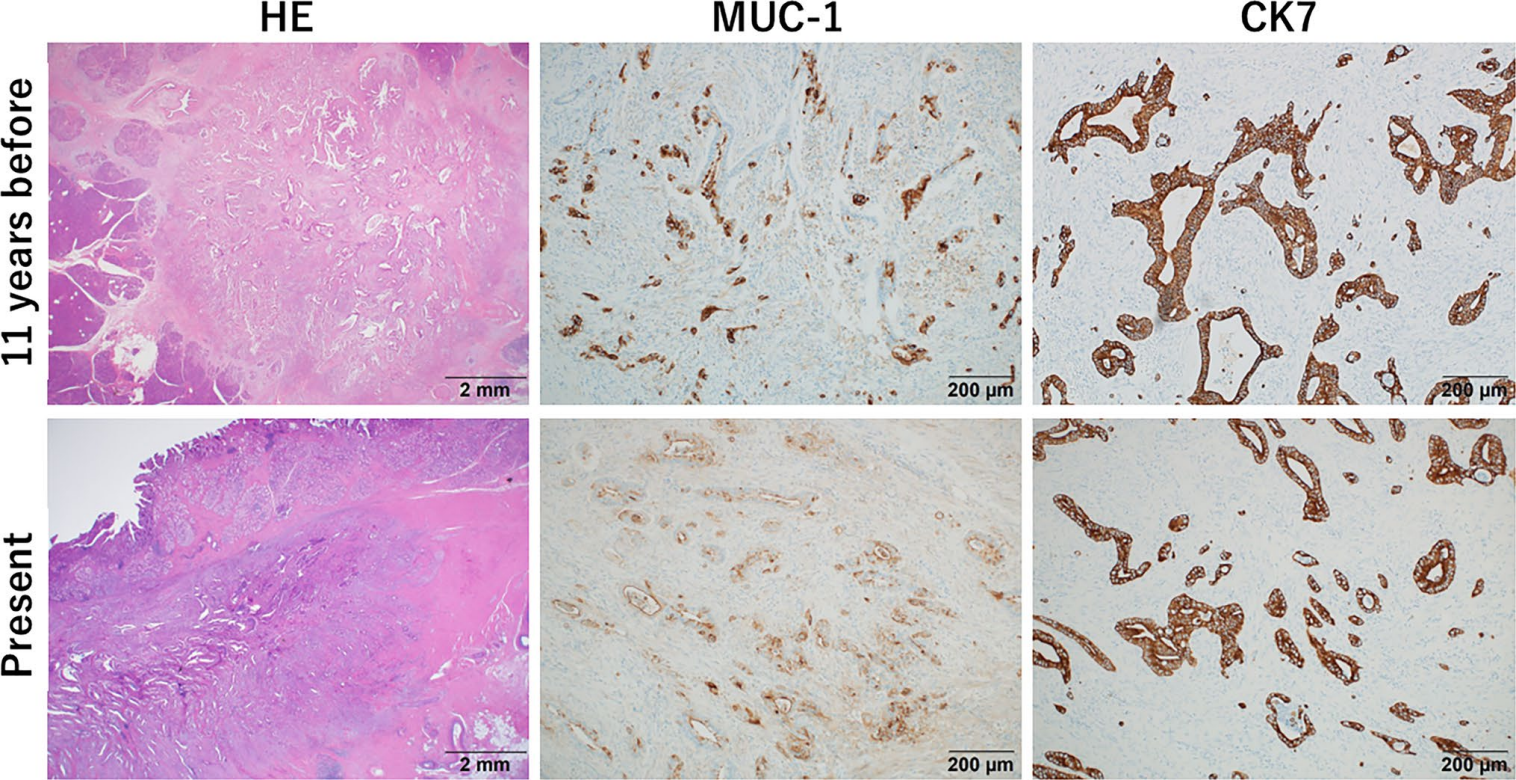
Histologic and immunohistochemical mucin (MUC) -1 and cytokeratin 7 (CK7) comparison between previous distal pancreatectomy (11 years before) and the present pancreatoduodenectomy. Histologically, both the previous and present specimens exhibited atypical tubular glands of varying sizes with fibrous desmoplasia. The morphologic features in the present case closely resembled those observed in the previous case. Immunohistochemically, adenocarcinoma cells from both specimens demonstrated positive staining for CK7 and MUC-1, indicating a consistent immunophenotypic pattern characteristic of pancreatic ductal adenocarcinoma

Earlier specimens showed well to moderately differentiated PDAC, which was morphologically similar to the present pathologic features. Immunohistochemistry analysis revealed positive staining for cytokeratin (CK) 7, mucin (MUC)-1, with partial positivity for CK20 and MUC-5AC. In contrast, CDX-2 (a homeobox gene encoding an intestine-specific transcription factor), MUC-2, and MUC-6 were negative. Based on the expression of these immunomarkers, intestinal differentiation of cancer cells was excluded. Rather, the observed immunopositive patterns for CK7 and CK20, as well as MUC-1 and MUC-5AC, were consistent with PDAC (Fig. 3). Furthermore, there was no mucosal lesions in the duodenum and the majority of cancer cells histologically occupied deeper submucosal layer of duodenal wall, while the stomach and pancreas remained intact. Thus, the final diagnosis was infiltrating adenocarcinoma in the duodenal wall, consistent with a late recurrence of PDAC.

The postoperative course was uneventful, and the patient was discharged from the hospital on postoperative day 8 with a grade I complication based on the Clavien–Dindo classification. In the present case, the patient received adjuvant therapy with four cycles of S-1 (80 mg/m^2^/day) and achieved 2 years disease-free survival.

## Discussion

This is the first report of intraduodenal wall recurrence 11 years after distal pancreatectomy for PDAC. There is no established definition of late recurrence in PDAC. Zang et al. defined late recurrence as occurring 12 months after surgery, while Luu et al. considered recurrences beyond approximately 5 years to be late. When considering long-term survivors who survived more than a decade, late recurrence of PDAC is very rare. Until now, only five cases have been reported, including the present case.

Dukmak et al. described a patient who had undergone a Whipple procedure with no adjuvant therapy 2 decades prior and remained cancer-free until a pulmonary nodule was identified. Pathologic examination of a lung mass biopsy specimen confirmed the presence of metastasis from the original pancreatic adenocarcinoma [3] (Table 1). Kitasato et al. reported the case of a 59-year-old woman who developed a solitary lung metastasis 13 years after distal pancreatectomy with adjuvant chemotherapy [4]. Masui et al. reported the case of a 57-year-old woman who underwent a Whipple procedure. Ten years later, a new lesion was found encasing the superior mesenteric artery. Uesato et al. reported the case of a 73-year-old man with two late recurrences at 4 and 11 years after pancreatoduodenectomy [6]. Although long-term survivors of more than decade are extremely rare, these reports emphasize that absence of disease at 5 years does not guarantee cure.

**Table 1:** Recurrence of pancreatic ductal adenocarcinoma in long-term survivors (more than a decade)

Author	Year	Age	Gender	Initial procedure	Pathology	Adjuvant therapy	Recurrence	Duration (y)	Tx	Second RFS (months)
Kitasato et al	2012	59	F	DP	Well	CDDP + 5FU + radiation	Lung	13	Resection	N/A
Masui T et al	2013	57	F	PD	Well	No	Para-aortic LN	10	Resection	12
Dukmak et al	2020	75	M	PD	N/A	No	Lung	20	Biopsy?	N/A
Uesato et al	2022	73	M	PD	Well	No	Lung	4 and 11	Resection	10
Present	2025	74	F	DP	Well to moderate	Gemcitabine	Duodenum wall	11	PD	24

*Tx* treatment, *RFS* recurrence free survival, *CDDP* cisplatin, *5FU* fluorouracil, *PD* pancreaticoduodenectomy, *DP* distal pancreatectomy, *LN* lymph node, *N/A* not applicable

In a multi-center study of 634 resected PDAC patients that focused on long-term survivors, investigators defined “late recurrence” as occurring > 1 year after surgery. They found that 39% of patients had late recurrence (beyond 1 year), whereas 44% had recurrence within the first year. None of the patients experienced a recurrence beyond 7 years of follow-up [1]. In cases of exceptionally late recurrence, the pattern often involves isolated pulmonary metastases rather than widespread disease. Patients with recurrence only in the lung tend to have longer disease-free intervals compared to those with recurrence in the liver or locoregional sites [7]. The present case is the first reported recurrence in the duodenal wall, but it is unclear whether post-recurrence survival is favorable.

Tai et al. described a 77-year-old woman with pancreatic head adenocarcinoma who developed synchronous multiple metastatic nodules in the duodenal mucosa. Biopsies confirmed that these duodenal lesions were metastases from PDAC [8]. However, there have been no reports of metachronous duodenal wall metastasis from PDAC originating in the pancreatic body or tail. Yamamoto et al. reported cases of PDAC metastasizing to other unusual gastrointestinal sites, which suggest the possibility of duodenal wall metastasis in the present case. In their report, two submucosal tumors in the stomach and one in the gallbladder developed 4 years after distal pancreatectomy for stage II PDAC and were completely resected by total gastrectomy and cholecystectomy [9]. They speculated a hematogenous pathway, as the metastatic lesions were located in the subserosa. Although the present case involved the duodenal wall, the recurrence pattern may be similar to their case.

Late-occurring recurrences, if isolated, can sometimes be managed aggressively. Reports have documented successful surgical re-resection of solitary recurrences (e.g., lung metastasectomy or locally recurrent tumor excision), leading to additional years of survival (Table 1). In the patient mentioned above with lung metastases discovered 13 years later, for example, the patient was still alive years after metastasectomy and ongoing chemotherapy [4]. Similarly, in the case of recurrence after 10 years, the mass was resected and the patient achieved disease control for a period thereafter [5]. These cases suggest that a very prolonged disease-free interval and a single, slow-growing recurrence may indicate that aggressive treatment of the recurrence is worthwhile. Further careful follow-up is required to monitor PDAC recurrence.

Oligometastasis has recently become a topic of interest in PDAC. Although there is no universal definition of “oligometastasis,” most studies use a maximum of 3–5 metastatic lesions as the cutoff, and a longer disease-free interval is generally considered to indicate more favorable tumor biology [10]. Patients who experience recurrence only in the lungs tend to have longer recurrence-free intervals and better survival compared to those with recurrence in the liver or peritoneum. A meta-analysis pooled data from 168 patients who underwent pulmonary resection for isolated metachronous PDAC metastases, demonstrating a 5-year survival rate of 37.0% [11]. The present case involved a solitary duodenal oligometastasis. Further accumulation of cases and long-term follow-up data is needed.

## Conclusion

We report the first case of late duodenum wall recurrence 11 years after distal pancreatectomy for PDAC.
